# C-752-01. Can ChatGPT-4o Handle the Heat? Benchmarking AI Against Clinicians in Burn Care: A Single-Blinded Study

**DOI:** 10.1093/jbcr/irag033.014

**Published:** 2026-04-06

**Authors:** Jia Lim, Poh (Leslie) Tan, David Sainsbury, Ben Strong, Sanjay Varma, Christopher J Lewis

**Affiliations:** Royal Victoria Infirmary, Newcastle, Newcastle upon Tyne, England; Royal Victoria Infirmary, Newcastle, Newcastle upon Tyne, England; Royal Victoria Infirmary, Newcastle, Newcastle upon Tyne, England; Cook University Hospital, Middlesbrough, England; Royal Victoria Infirmary, Newcastle, Newcastle upon Tyne, England; Northern Regional Burn Centre, Newcastle Upon Tyne, England

## Abstract

**Introduction:**

Artificial intelligence (AI) tools such as ChatGPT are increasingly used by the public to obtain health advice. However, their accuracy in acute burn care advice remains uncertain. This study aimed to assess the domain-specific accuracy and quality of ChatGPT’s burn guidance in comparison to clinician responses, using British Burn Association (BBA) guidelines as the benchmark.

**Methods:**

A single-blinded, cross-sectional comparative study was conducted using 20 burn scenarios of varying severities, burn types, and patient groups. Clinicians’ and ChatGPT’s responses were blinded and independently evaluated by burns Consultants across five domains: first aid, dressing, pain relief, referral, and safety warnings. Correctness was scored as 1 (correct) or 0 (incorrect) according to BBA recommendations, with discrepancies resolved by a third reviewer. Overall response quality was assessed using a modified Global Quality Score (mGQS; 1–5), with scores ≥4 considered clinically acceptable. McNemar and paired t-tests compared domain accuracy and mean mGQS respectively.

**Results:**

Clinicians demonstrated higher overall domain accuracy (88%) compared to ChatGPT (78%). Performance was comparable in first aid (85% each) and referral (100% each), with ChatGPT showing marginally lower accuracy in dressing (85% vs 90%) and safety (90% vs 100%). Pain relief accuracy was notably lower for ChatGPT (30% vs 65%, *p* ≈ 0.023). Mean mGQS scores were higher for clinicians (4.33 ± 0.69 vs 4.15 ± 0.63), with 80% versus 70% of responses judged clinically acceptable; however, these differences did not reach statistical significance.

**Conclusions:**

ChatGPT provides generally safe and understandable initial burn guidance, performing comparably to clinicians in high-priority domains such as first aid, referral, and safety. While accuracy was lower for pain relief guidance, this largely reflects minor gaps in advice rather than critical errors. These findings suggest that AI tools like ChatGPT can offer reliable immediate support to the public while appropriately emphasising the need for professional evaluation when required.

**Applicability of Research to Practice:**

AI models can provide burn management guidance with accuracy comparable to clinicians in key domains. Their utility lies in first aid, referral, and safety support when timely professional input is unavailable. However, further validation across diverse burn scenarios is required to improve reliability. Clear disclaimers remain essential to ensure users recognise limitations and do not assume certainty. With continued refinement and transparent communication, AI can complement clinician-led care, offering a valuable adjunct in community burn first aid and triage.

**Funding for the study:**

N/A.

